# Modelling of Effective Thermal Conductivity of Composites Filled with Core-Shell Fillers

**DOI:** 10.3390/ma13235480

**Published:** 2020-12-01

**Authors:** Jan Czyzewski, Andrzej Rybak, Karolina Gaska, Robert Sekula, Czeslaw Kapusta

**Affiliations:** 1 Hitachi ABB Power Grids Research, 31-154 Krakow, Poland; robert.sekula@hitachi-powergrids.com; 2 ABB Corporate Technology Center, 31-038 Krakow, Poland; andrzej.rybak@pl.abb.com; 3 Department of Solid State Physics, Faculty of Physics and Applied Computer Science, AGH University of Science and Technology, 30-059 Krakow, Poland; karolina.gaska@bristol.ac.uk (K.G.); kapusta@agh.edu.pl (C.K.); 4 Department of Aerospace Engineering, University of Bristol, Bristol BS8 1TR, UK

**Keywords:** polymer-matrix composite, epoxy resin, filled epoxy, core-shell filler, thermal properties, effective thermal conductivity, analytical modelling, boron nitride, cellulose

## Abstract

An effective model to calculate thermal conductivity of polymer composites using core-shell fillers is presented, wherein a core material of filler grains is covered by a layer of a high-thermal-conductivity (HTC) material. Such fillers can provide a significant increase of the composite thermal conductivity by an addition of a small amount of the HTC material. The model employs the Lewis-Nielsen formula describing filled systems. The effective thermal conductivity of the core-shell filler grains is calculated using the Russel model for porous materials. Modelling results are compared with recent measurements made on composites filled with cellulose microbeads coated with hexagonal boron nitride (h-BN) platelets and good agreement is demonstrated. Comparison with measurements made on epoxy composites, using silver-coated glass spheres as a filler, is also provided. It is demonstrated how the modelling procedure can improve understanding of properties of materials and structures used and mechanisms of thermal conduction within the composite.

## 1. Introduction

Heat management is one of the key challenges in electronic and power devices. Effective heat dissipation is needed to achieve required ratings. This, in turn, requires high thermal conductivity of the applied electric insulation, structural, and packaging materials [1]. Filled epoxy resin composites are often used in such applications and enhancement of thermal conductivity of such composites is thus of high interest [2].

Thermal conductivity of composites has been extensively studied and it depends on that of the polymer matrix, that of the filler material, filling degree, filler grain shape, and their mutual arrangement. An extensive review is given, for example, in References [3] and [4]. The filler-matrix interfacial thermal resistance can also influence the thermal conductivity [5]. This is important at cryogenic temperatures [6], and for nano-fillers, also at room temperature [7]. Electrically insulating fillers of high thermal conductivity are of particular interest since they can improve the thermal conductivity of the composite material, while maintaining its electrically insulating properties. Aluminium nitride [8] and boron nitride [9] are most often quoted in relation to such applications. Due to the high cost of such fillers and viscosity rise of the processed material at increasing filler load, it is desirable to achieve the thermal conductivity enhancement while keeping the filler content at a possibly low level. Various methods reaching to that goal have been proposed. Hybrid fillers, e.g., mixtures of fillers with different shapes, such as plate-like shaped, spherical particles, or rods mixed together, can create efficient conductive paths. An example of a recent study is given in Reference [10] for a hybrid system of graphene nanofiller and anthracite microfiller. Other examples include various spherical and fibrous microfillers studied in Reference [11], boron nitride–silica and aluminium nitride–silica hybrid systems in Reference [12], and a graphene nanoplatelet-silica system in Reference [13].

Another promising approach is application of filler materials of core-shell structure, wherein filler grains are composed of a low thermal conductivity core with an outer shell of a high-thermal-conductivity (HTC) material. An early study of thermal conductivity of polyethylene filled with metal-coated polyamide particles is given in Reference [14]. Enhanced thermal conductivity, dielectric, and thermo-mechanical properties of epoxy composites filled with core-shell silica-reduced-graphene-oxide structures was demonstrated in Reference [15]. Another example of application of graphene as a HTC material for a core-shell filler is given in Reference [16] with the enhancement of the thermal conductivity much larger than with pristine graphene nanosheets. Enhanced thermal conductivity of epoxy composites with core-shell fillers with electrically insulating HTC material was demonstrated in Reference [17] with aluminium-nitride-coated alumina and silicon-nitride-coated silica, and in Reference [18] for boron-nitride-coated silica fillers. An extensive set of measurements of a composite employing cellulose beads with a boron nitride shell was also recently reported in Reference [19].

As the application of core-shell fillers draws increasing attention, a proper model to predict the effective thermal conductivity is needed. An example of numerical calculation of the effective thermal conductivity of such composites, based on finite element method simulations, can be found in References [20] and [21]. These can describe the nature of thermal conductivity in such systems and can bring understanding of its influential factors but require a complex procedure to build and analyse material models. There is a lack of a simple effective model which could allow material engineers to assess orders of magnitude of thermal conductivity of a composite with a core-shell-type filler, based on the properties of the applied materials.

In the present work, an effective model of thermal conductivity of composite materials with core-shell fillers is proposed. The model employs the Lewis-Nielsen effective model of thermal conductivity of a filled system, wherein the thermal conductivity of the filler is significantly larger than that of the matrix material surrounding the filler [22]. The effective thermal conductivity of the core-shell filler grains themselves is calculated using the Russel model [23], which describes thermal conductivity of porous materials. A detailed explanation as to why this model is applicable is given later in this paper.

With regard to the Lewis-Nielsen model, its advantage is that it parametrizes in a simple way both the effect of anisotropic shapes and arrangement of the filler grains and the effect of interaction of the grains when the filler load approaches the maximal content level for a particular system. Other models, such as, e.g., the Cheng-Vachon model with the Okamoto-Ishida modification [24], or the Agari-Uno model [25], could also be used. However, the Lewis-Nielsen model was chosen since its parameters have the most intuitive meaning. The theoretical values of these parameters, for a large number of filler shapes, were calculated and are given in Reference [26]. The meaning of these parameters, in particular the maximal filler concentration value, was also discussed in Reference [27], where it was demonstrated that its experimental value is more relevant to be used, compared to the theoretical values which can only describe idealized, not fully realistic systems. The physical notion of the Lewis-Nielsen model allowed the authors of Reference [7] to extend it to systems with a thermal resistance of the filler-matrix interface in nanocomposites, and it was shown that the model can describe experimental measurements very well. Another interesting piece of validation of the Lewis-Nielsen model is given in Reference [28], where it is confronted with finite-element-method calculation of thermal conductivity of composites with various filler shapes and good agreement is obtained.

The model calculations are compared to our experimental measurements made on a model system of spherical glass beads which were used as core material and were coated with a silver shell. A set of samples based on epoxy resin, filled with such a filler, was prepared and compared with reference samples with uncoated beads. The model calculations are also compared to an extensive set of measurements reported in Reference [19] and thoroughly analysed.

## 2. Materials and Methods

### 2.1. Model of Thermal Conductivity

Composite material with a core-shell filler is a complex system, because the filler itself consists of two materials of strongly differing thermal conductivities. The presented model of the thermal conductivity of such a composite consists of the following parts:

- Calculation of the effective thermal conductivity of the core-shell filler grain.
- Calculation of the thermal conductivity of the matrix-filler composite with the filler of the given effective thermal conductivity.

#### 2.1.1. Effective Thermal Conductivity of Coated Filler

**Figure 1:**
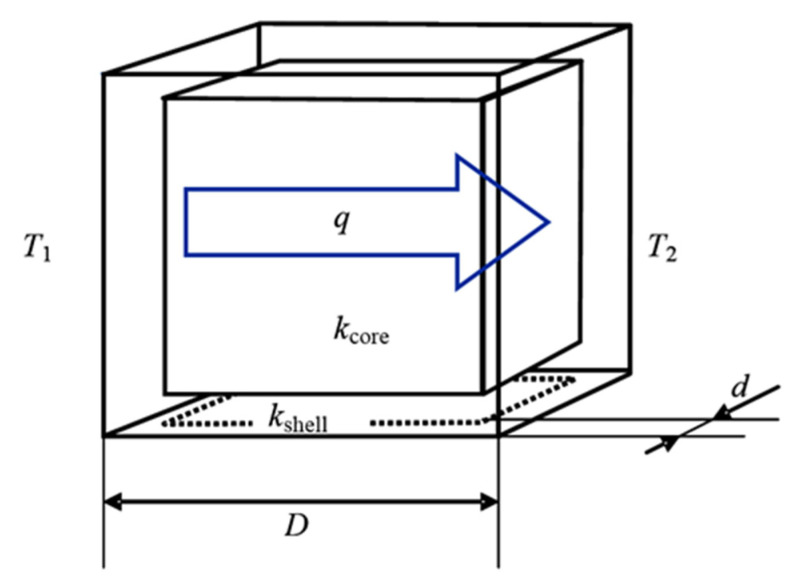
Simplified model of the core-shell filler grain. The heat flow q runs from the left wall, of temperature $T_{1}$, to the right one, of the temperature $T_{2}$. In case the thermal conductivity of the shell is much larger than that of the core, the heat flux is transported on the major part through the shell material and on the smaller part, in parallel, through the core (parallel model). The part transported through the core meets on its path the core material of thickness D−2d and that of the shell of thickness of 2d (series model).

The filler grains with HTC shell were treated as a system of two materials with very different thermal conductivities. Such a system is very different from typical 2-phase systems in which the HTC phase is randomly dispersed in the low-conductivity matrix. In a simplified approach, one can model the core-shell grain by a cube, as shown in Figure 1. This is not meant as a system corresponding to the realistic geometry in physical composites, but it is only used to explain the basic physical phenomena in the thermal conduction of a core-shell structure. The cube side length is D and the HTC shell thickness is d. The volume fraction of the shell in the grain is:

(1) $$\varphi_{\mathrm{shell}}=1-{\left(1-\frac{2d}{D}\right)}^{3}$$

For small thickness of the shell, and, thus, small HTC fraction, $\varphi_{\mathrm{shell}}$, the effective thermal conductivity, $k_{\mathrm{eff}\;\mathrm{grain}},$ of the filler grain can be calculated as an appropriate combination of the parallel and the series models [29] of thermal conductivity of composite systems:

(2) $$k_{\mathrm{eff}\;\mathrm{grain}}\approx \frac{2}{3}\varphi_{\mathrm{shell}}k_{\mathrm{shell}}+\left(1-\frac{2}{3}\varphi_{\mathrm{shell}}\right){\left(\frac{\varphi_{\mathrm{shell}}}{3k_{\mathrm{shell}}}+\frac{3-\varphi_{\mathrm{shell}}}{3k_{\mathrm{core}}}\right)}^{-1}$$

(3) $$\approx \frac{\left(3-2\varphi_{\mathrm{shell}}\right)k_{\mathrm{core}}+2\varphi_{\mathrm{shell}}k_{\mathrm{shell}}}{\varphi_{\mathrm{shell}}k_{\mathrm{core}}+\left(3-\varphi_{\mathrm{shell}}\right)k_{\mathrm{shell}}}k_{\mathrm{shell}}$$

where $k_{\mathrm{shell}}$ and $k_{\mathrm{core}}$ are the thermal conductivities of the shell and the core, respectively. The approximations hold for small $\varphi_{\mathrm{shell}}$. The formula in Equation (3) appears to be a special case of the Russel model [23] of thermal conductivity of porous materials with matrix material of conductivity, $k_{\mathrm{shell}},$ and pore-filling material conductivity, $k_{\mathrm{core}},$ at the limit of small volume fraction of the matrix with respect to the pores. The Russel model describes spherical inclusions in the porous material and is better suited than that of Figure 1 to describe core-shell grains. This is the reason why it was chosen to be used here. The Russel formula reads:

(4) $$k_{\mathrm{eff}\;\mathrm{grain}}=\frac{{\left(1-\varphi_{\mathrm{shell}}\right)}^{2/3}k_{\mathrm{core}}+\left[1-{\left(1-\varphi_{\mathrm{shell}}\right)}^{2/3}\right]k_{\mathrm{shell}}}{\left[{\left(1-\varphi_{\mathrm{shell}}\right)}^{2/3}-\left(1-\varphi_{\mathrm{shell}}\right)\right]k_{\mathrm{core}}+\left[2-\varphi_{\mathrm{shell}}-{\left(1-\varphi_{\mathrm{shell}}\right)}^{2/3}\right]k_{\mathrm{shell}}}k_{\mathrm{shell}}$$

$\varphi_{\mathrm{shell}}$ can also be expressed by the shell thickness using Equation (1), which also holds for spherical grains:

(5) $$k_{\mathrm{eff}\;\mathrm{grain}}=\frac{{\left(1-2\frac{d}{D}\right)}^{2}k_{\mathrm{core}}+4\frac{d}{D}\left(1-\frac{d}{D}\right)k_{\mathrm{shell}}}{2\frac{d}{D}{\left(1-2\frac{d}{D}\right)}^{2}k_{\mathrm{core}}+\left(1-2\frac{d}{D}+8{\left(\frac{d}{D}\right)}^{2}\left(1-\frac{d}{D}\right)\right)k_{\mathrm{shell}}}k_{\mathrm{shell}}$$

Unlike the formula in Equation (2), the formulae of Equations (3) and (4) approach the value of $k_{\mathrm{shell}}$ at the limit of $\varphi_{\mathrm{shell}}$ approaching 1, and thus, they describe the effective thermal conductivity of the filler well, also for large thickness of the shell. The equivalence of the models of Equations (2) and (4) at small $\varphi_{\mathrm{shell}}$ means that, in this limit, the Russel model is a good approximation also for not spherical fillers, wherein the filler grain dimensions are equal in all directions (aspect ratio equal to 1). The novelty of this approach is in the finding that the Russel formula, originally developed to describe a continuous porous material, can also be used to describe the effective thermal conductivity of a single core-shell filler grain.

**Figure 2:**
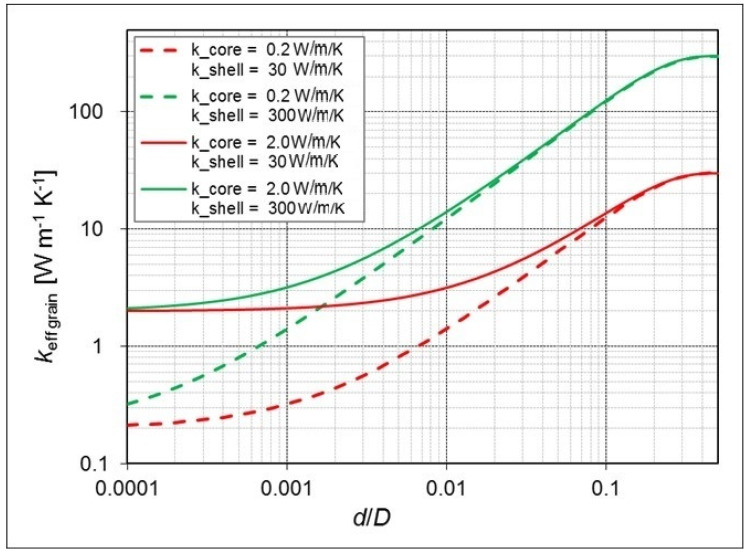
Effective thermal conductivity of a core-shell filler according to Equation (4), plotted versus the ratio of the thickness of the shell d to the total grain diameter D, for the core thermal conductivities $k_{\mathrm{core}}$ of 0.2 and 2.0 W m^–1^K^–1^ and the shell conductivities $k_{\mathrm{shell}}$ of 30 and 300 W m^−1^K^−1^.

The dependence of $k_{\mathrm{eff}\;\mathrm{grain}}$ on d/D according to the Russel model calculated using Equation (5) is shown in Figure 2 for two example values of $k_{\mathrm{core}}$ and two values of $k_{\mathrm{shell}}$. A high thermal conductivity of the shell is needed for a significant effect on that of the core-shell grain. Then, even at small shell thickness, the influence of the thermal conductivity of the core has a marginal meaning, and the main function of the core becomes only to mechanically support the conductive shell.

#### 2.1.2. Model of Thermal Conductivity for Fixed Size of Core-Shell Grain

Having calculated the effective thermal conductivity of the core-shell filler grain, one can calculate the conductivity of the epoxy-matrix composite using one of the known models. As discussed in Section 1, the Lewis-Nielsen model [22] was chosen in which the effective thermal conductivity of the composite equals:

(6) $$k_{\mathrm{eff}}\left(\varphi \right)=\frac{k_{f}+Ak_{m}+A\left(k_{f}-k_{m}\right)\varphi }{k_{f}+Ak_{m}-\left(1+\frac{1-\varphi_{\max}}{\varphi_{\max}}\;\frac{\varphi }{\varphi_{\max}}\right)\left(k_{f}-k_{m}\right)\varphi }k_{m}$$

where φ is the filler volume concentration and $k_{m}$ and $k_{f}$ are the thermal conductivities of the matrix and the filler, respectively. For the core-shell filler, the filler thermal conductivity $k_{f}$ has to be replaced by $k_{\mathrm{eff}\;\mathrm{grain}}$. The model has two parameters:

- Limiting filler concentration $\varphi_{\max}$ corresponding to the maximally packed filler,
- Parameter *A* depending on the shape, positioning, and orientation of the filler grains.

The limiting value of thermal conductivity with maximally packed filler, $\varphi =\varphi_{\max}$, equals:

(7) $$k_{\mathrm{eff}}\left(\varphi_{\max}\right)=\frac{\left(A\varphi_{\max}+1\right)\;k_{f}+A\left(1-\varphi_{\max}\right)k_{m}}{A+1}\;$$

which at large values of A approaches the limit of the parallel conductivity model, the highest bound for the conductivity of a two-phase system with $k_{f}>k_{m}$ [29]. At A=0 and $\varphi_{\max}=1,$ the effective thermal conductivity, $k_{\mathrm{eff}}\left(\varphi \right),$ coincides with the series conductivity model [29], which is a lower bound for the system, see also Reference [7].

Theoretically calculated values of the parameters $\varphi_{\max}$ and A for different filler shapes and orientations are summarized in Reference [26]. For an ideal system of interacting (not overlapping) spheres, these parameters are $\varphi_{\mathrm{ma}x}=0.637$ and $A=2.5/\varphi_{\max}-1\approx 2.92$. In this case, the Lewis-Nielsen model is an extension of the Bruggeman model of interacting spheres [30] and the two models coincide for φ smaller than around 0.4. For higher concentrations, the effect of the limited concentration makes the conductivity in the Lewis-Nielsen model rise faster than for the Bruggeman model.

In general, the parameter A depends on two factors:

- The filler grain shape, mainly the aspect ratio L/l of the filler grains, where L is its largest dimension and l the smallest one; the larger the aspect ratio, the larger A.
- The randomness of positioning of the filler particles, both in the distances between the particles and in their mutual angular orientation.

As an example, for an ideal system of randomly distributed rods of the aspect ratio L/l, the parameter A is of the order of L/(2l) and for uniaxially oriented rods, for the conduction direction parallel to the rods, it equals 2L/l, while in the direction perpendicular to the rods, it is only 0.5 [26]. In the oriented case, for the conduction direction parallel to the rods, A is around 4 times larger compared to the random case just due to the ordered orientation of the rods. But at the same time, for a large aspect ratio, the value of A of the random case is still significantly larger than that of the oriented case for the conduction direction perpendicular to the rods.

Similar to the case of high-aspect-ratio fillers, one can expect the parameter A to be larger where particles of small aspect ratio tend to come in contact with each other due to local anisotropic interaction forces and tend to form chains. In both cases, the randomness of distances and orientation of the filler particles is broken, leading to an increase of A, at least in particular directions of conduction.

The theoretical values of $\varphi_{\max}$ are also given in Reference [26], for systems with identical particles of defined shapes. However, as shown in Reference [27], for example, the values of $\varphi_{\max}$ should rather be treated as effectively maximal values, ideally being the experimentally measured limiting values, which normally depend not only on the filler shape but also on many other factors, such as the mixing procedure, filler surface functionalisation, viscosity of the matrix during mixing, etc.

All these features make the Lewis-Nielsen model an effective tool, making it possible to assess various characteristics of the composite, based on the measurement of the thermal conductivity and its dependence on the filler concentration.

#### 2.1.3. Influence of Distributed Grain Size

For a core-shell filler with a fixed shell thickness, which is the typical case for most of the manufacturing methods, the thermal conductivity of the filler depends on the filler grain size. For example, if a 1 μm HTC thickness is applied on a grain of 3 μm diameter, the effective thermal conductivity of the grain will be much higher compared to a grain of 100 μm diameter and the same HTC shell thickness. For a core-shell filler with a wide grain size distribution, application of the model described in Section 2.1.1 and Section 2.1.2 requires taking an average of the effective thermal conductivity, weighted by the percentage contributions from each size fraction. Such an approach was adopted in this paper. One can build a model with filler sizes divided in fractions, but we verified that, for a continuously distributed filler size, such an approach gives virtually the same result as with the average of conductivity from all sizes.

### 2.2. Experimental

In this section, the core-shell filler composites made with silver-coated glass microspheres are described. The main purpose of this experiment was a quick check of how much the thermal conductivity can be increased by adding a high-thermal-conductivity shell to a core of moderate thermal conductivity, but also significantly larger than that of the matrix polymer.

#### 2.2.1. Sample Preparation Process

Epoxy resin derived from bisphenol A (DGEBA) CY 228-1, an anhydride hardener HY918, a flexibiliser DY 045, and an amine accelerator DY 062, all from Huntsman (Huntsman Advanced Materials (Europe) BVBA, Everberg Belgium), were used. All ingredients were mixed in the ratio CY228-1 100 pbw, HY918 80 pbw, DY 045 20 pbw, and DY 062 1.5 pbw, and stirred. Then, the filler particles were introduced and stirred for 15 min. Samples were cast into silicone moulds and degassed in vacuum at 2 mbar pressure to remove trapped air followed by curing for 4 h at 80 °C and 8 h at 140 °C.

Customised silver-coated glass microspheres delivered by Brazel Technology (Brazel Technology GmbH, Kirchheim, Germany), SG1-32139, with the nominal silver shell thickness of 1 μm, were used. Samples were manufactured with 31% and 45% filler volume content. Uncoated glass microspheres, SG1-32130, were used as the reference filler. For a metal film of thickness of 1 μm, the thermal conductivity is at the level of its bulk-material value. As it was shown in Reference [31], the finite-size effects for copper occur for film thickness smaller than ca. 4 nm and similar values can be expected for silver.

None of the tested fillers was possible to be mixed in the epoxy matrix to the volume concentration larger than around 31%. Above that level, the processability of the composites was hindered due to a very high viscosity of the mixture and problems with the degassing of the sample. In order to test larger concentrations, the filler grain surface was chemically functionalized with silane. Modification of the filler surface was done by dipping in a diluted aqueous solution of Z-6020 silane from Dow Corning (0.2% silane concentration). The silane solution was acidified to a pH of 4.0 with acetic acid to obtain optimum performance of filler material. After applying the silane, the glass spheres were dried at 120 °C for complete condensation of silanol groups at the surface and to remove water. Due to the silane treatment, it was possible to achieve the volume concentration of 45% for both the reference and the core-shell fillers.

#### 2.2.2. Measurements

Thermal conductivity was measured using a PPMS (Physical Property Measurement System) device from Quantum Design Inc. (San Diego, CA, USA). Cylindrical specimens, with the diameter of 6 mm, were cut and polished. Heat pulses were applied by the current running through copper electrodes, which were spliced to the opposite bases of the samples. The thermal conductivity measurements were performed in high vacuum. The control software of PPMS uses a special algorithm to optimize measurement parameters, such as heat pulse duration, heater current, and frequency [32].

Morphology of the filler particles and the composites was studied by means of scanning electron microscopy (SEM), using Nova NanoSEM 200 (FEI, Hillsboro, OR, USA).

## 3. Results and Discussion

### 3.1. Example of Core-Shell Filler Composite

**Figure 3:**
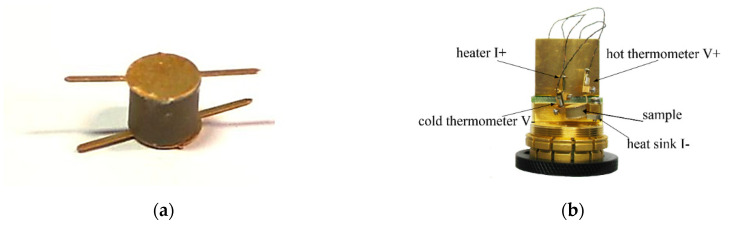
Macroscopic pictures of samples of obtained composites: (**a**) Sample of 6 mm diameter assembled with copper electrodes and (**b**) sample assembled in the PPMS (Physical Property Measurement System).

In this section, properties and thermal conductivity measurement results of an epoxy-resin-based composite filled with sliver-coated glass core-shell microbeads are described. Results for uncoated beads treated as a reference are also given. A sample of the obtained composite used for thermal conductivity measurements is shown in Figure 3a. Figure 3b shows the sample assembled in the thermal conductivity measurement system holder.

#### 3.1.1. Filler Morphology

**Figure 4:**
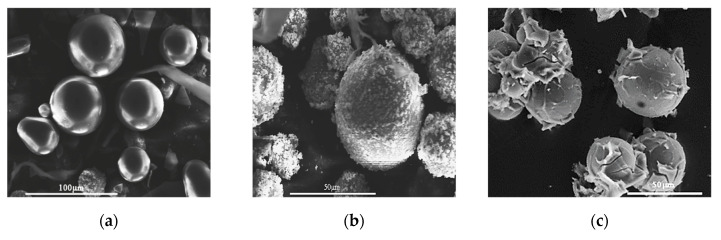
Scanning electron microscope (SEM) observation result for: (**a**) The core glass bead reference filler material, (**b**) the core-shell filler, and (**c**) the glass bead reference filler after silane treatment.

**Figure 5:**
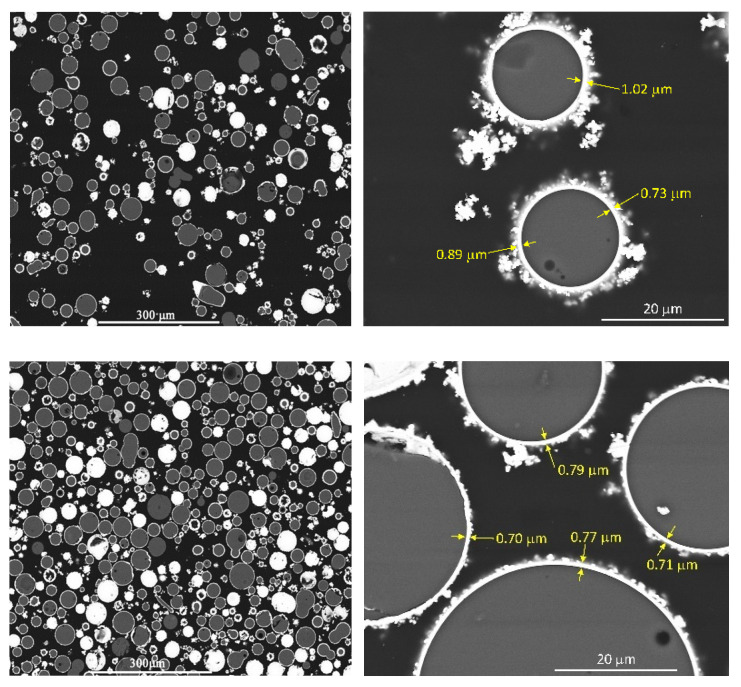
SEM micrographs with different magnification for the composite of 31% volume content (**top**) and 45% (**bottom**) of silver-coated glass beads dispersed in the epoxy matrix.

**Figure 6:**
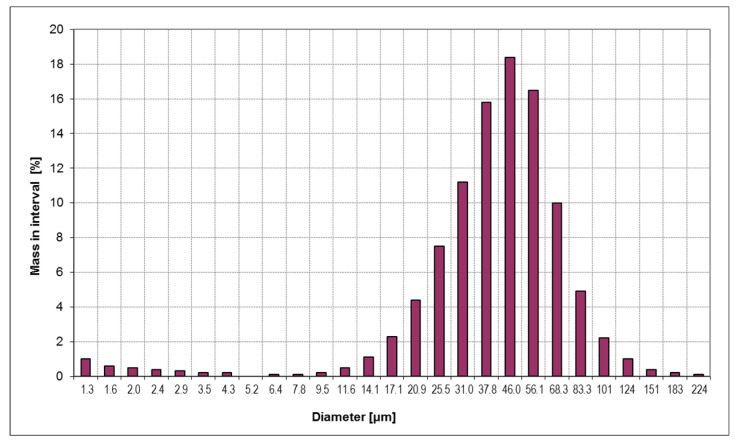
Particle size distribution of the core-shell filler.

The pure glass microspheres are presented in Figure 4a, where the smooth surface of the uncoated filler can be seen. In Figure 4b, the silver coating (shell) can be observed with the irregular and rough surface. The filler after the surface treatment with silane is presented in Figure 4c. Additionally, SEM observations were made for the cut and polished samples of the composites, with 31% and 45% volume content of the filler, and are presented in Figure 5. The microscopic observations revealed that the shell thickness was on average 0.8 μm, very close to the value of 1 μm stated by the supplier. The grain size distribution of core-shell filler was measured and is presented in Figure 6.

#### 3.1.2. Thermal Conductivity Measurements

**Figure 7:**
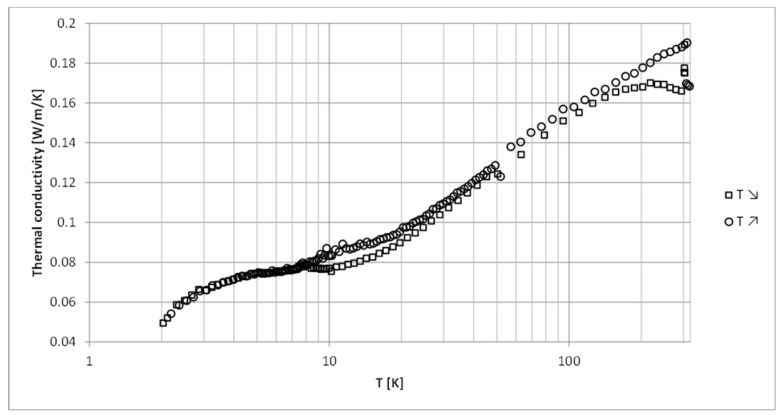
Thermal conductivity of pure epoxy resin measured down to the temperature of 2 K. Squares and circles correspond to temperature decrease and increase, respectively.

In order to check consistency of the measurements, the thermal conductivity of the pure epoxy resin was measured down to cryogenic temperatures. The results are shown in Figure 7. They feature a plateau between 4 and 10 K at the level of 0.075 ${\mathrm{Wm}}^{-1}K^{-1}$, characteristic for amorphous materials, with conductivity of 0.05 ${\mathrm{Wm}}^{-1}K^{-1}$ at 2 K and around 0.18 ${\mathrm{Wm}}^{-1}K^{-1}$ at room temperature. This reproduces the results found earlier in References [6] and [33]. Some fluctuations of the measured values at room temperature can be seen, as well as a discrepancy between the values read during the cooling and the reheating of the sample. As it was not possible to eliminate those, they were assigned to measurement errors, which were judged to be of the order of 10%. This error value was thus assigned to all our measurements.

**Figure 8:**
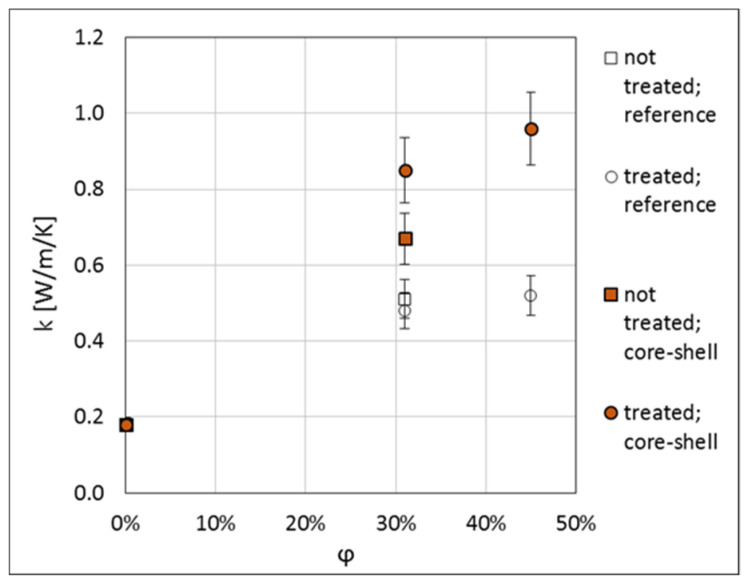
Thermal conductivity results for composites filled with core-shell (filled points) and reference fillers (open points). Squares correspond to the fillers without surface treatment, circles to the fillers treated with silane. Measurement errors of 10% are assumed (see text).

The measured thermal conductivities of the produced composites are presented in Table 1 and in Figure 8. For the 31 vol.% composite with the untreated core-shell filler, the thermal conductivity is increased by about 30% with respect to the untreated reference filler. For the silane-treated fillers, the increase of the thermal conductivity of the core-shell filler with respect to the reference filler is by 77% and 84% for the concentrations of 31 and 45 vol.%, respectively. This result shows that application of core-shell fillers is an efficient method for enhancement of the thermal conductivity of polymer composites while minimising the amount of the HTC material used, thus confirming the earlier results of References [14,15,16,17,18,19].

### 3.2. Comparison of Experimental Results to the Developed Model

#### 3.2.1. Silver-Coated Glass Beads

The experimental results of Section 3.1 were compared to the model described in Section 2.1 with $k_{m}$ = 0.18 ${\mathrm{Wm}}^{-1}K^{-1}$, $k_{\mathrm{core}}$ = 1.38 ${\mathrm{Wm}}^{-1}K^{-1}$, and $k_{\mathrm{shell}}$ = 420 ${\mathrm{Wm}}^{-1}K^{-1}$. The epoxy matrix thermal conductivity was taken from our measurement, shown in Section 3.1.2, that of the core is the typical value for fused silica [34], and that of the shell is that of metallic silver. The resulting average effective thermal conductivity of the core-shell filler grains, with the shell thickness of 0.8 μm, and with the uncoated grain size distribution of Figure 6, is 48.7 ${\mathrm{Wm}}^{-1}K^{-1}$. The average shell thickness to diameter ratio is d/D = 0.034.

**Figure 9:**
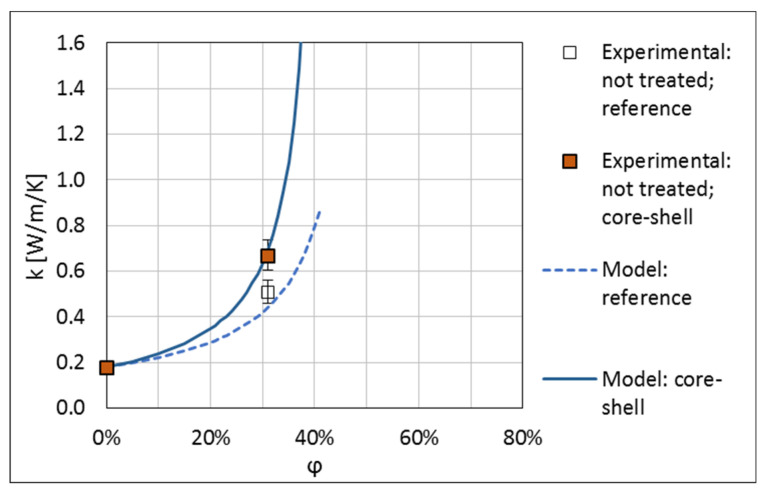
Thermal conductivity of composites with untreated fillers. The lines show the results of model calculations.

**Figure 10:**
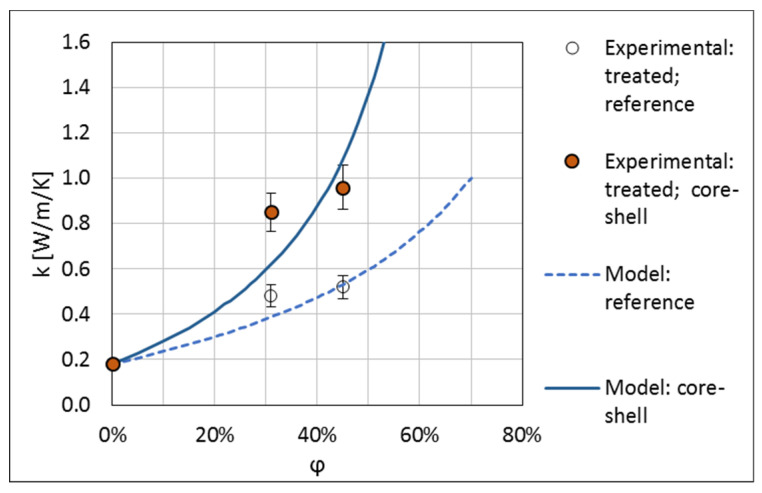
Thermal conductivity of composites with fillers treated with silane. The lines show the results of model calculations.

Figure 9 and Figure 10 show the thermal conductivity of composites with the untreated and treated fillers respectively, compared with the described model. The results for both the reference filler and the core-shell filler are shown. Parameters of the model fitted to the experiment are given in Table 2. For the fit, A was kept not smaller than 1.5, i.e., at the case of not interacting spheres [26], a reasonable physical bound. The maximum filler content was kept not larger than that for the hexagonal-close packing of spheres, i.e., $\varphi_{\max}\leq 74.05\%$ [26].

The number of the experimental points is small but, even with this, one can draw some conclusions form the model fit. For the untreated filler, the lowest limiting value of 1.5 for the parameter A was obtained. This value corresponds to randomly distributed spheres of identical size, which do not interact, i.e., can overlap, which is not a physical assumption. We checked that the theoretical parameter values for interacting, not overlapping spheres, $A=2.5/(\varphi_{\max}-1)$, gave a much worse fit. However, one has to bear in mind that, with the broad distribution of the diameters of the filler particles, the effect of interaction of the spheres starts at a significantly higher concentration than for spheres of identical size.

Conversely, relatively large values of A, somewhat larger than the value for interacting spheres, were obtained for the treated filler. This may be related to aggregation of filler grains into larger structures due to silanization, as is visible in Figure 4c. This leads to an effective increase of the aspect ratio of the filler particles and, thus, to an increase of the parameter A. A similar effect of aggregation of the filler grains, manifesting itself in large values of the parameter A obtained in fitting the thermal conductivity data, was observed in Reference [7].

The maximal concentration values obtained reflect the experimental observations made. For the untreated filler, $\varphi_{\max}$ is about 40%, close to the limit encountered in the composite mixing. For the treated filler, which mixes much better, high values of $\varphi_{\max}$ are obtained. This shows that, when using the Lewis-Nielsen model, the maximal filler concentrations, $\varphi_{\max}$, should be interpreted as those resulting from the processing nature of the systems considered and not as the theoretical values for a particular shape of the filler grains. This confirms the validity of the approach proposed in Reference [27].

The above indicates that the methodology with fitting both the A parameter and $\varphi_{\max\;}$, with their values bound by the allowed theoretical limits, is the right way to apply the Lewis-Nielsen model, unlike an often-adopted approach, in which the theoretical values merely based on the shape of the filler grains are taken for the calculation.

#### 3.2.2. Boron-Nitride-Coated Cellulose Beads

In this section, the thermal conductivities of epoxy composite systems, with a core-shell filler consisting of cellulose beads coated with platelets of hexagonal boron nitride (h-BN), as reported in Reference [19], are discussed. These experimental results are compared to the model described in Section 2.1.

Two sizes of platelets were tested in Reference [19], 8 and 18 μm, labelled BN-S and BN-L, respectively. The core-shell microbeads were produced in three grades, two with BN-S, labelled Cell-1 and Cell-2, of a diameter of ca. 30 and ca. 40 μm respectively, and one with BN-L, labelled Cell-3 of a diameter of ca. 35 μm.

Composites with three different filler concentration levels for each of the filler grades were produced by a compression moulding process. For comparison, samples of epoxy filled with pristine h-BN were made, covering both the BN-S and the BN-L grades. Thermal conductivity of all the samples was measured in both the thickness and in-plane directions.

For our modelling purposes, we calculated the effective thermal conductivity, $k_{\mathrm{eff}\;\mathrm{grain}},$ of the filler according to Equation (4), using the concentration by mass of h-BN in the core-shell beads given in Reference [19]. To convert into the volume concentration, we took the density of cellulose of 1.5 g/cm^3^ [35], and that of h-BN of 2.2 g/cm^3^ [36]. The thermal conductivity of the core $k_{\mathrm{core}}=0.23\;{W\;m}^{-1}K^{-1}$, for densely packed cellulose [37], was assumed. The thermal conductivity of the shell is the effective in-plane thermal conductivity of a structure formed by the h-BN platelets surrounding the cellulose core. As shown in microscopic images in Reference [19], these tend to be positioned parallel to the surface of the core, so the shell thermal conductivity should be determined by the in-plane conductivity of h-BN and the conductivity of interfaces between the platelets. In the case of BN-L/Cell-3, the size of the platelets is only slightly smaller than the diameter of the core, so the influence of the interfaces may be expected to be small and the shell thermal conductivity comparable to the conductivity of h-BN may be expected. The thermal conductivity of h-BN is extremely anisotropic with the conductivity of the order of 250 to 500 ${\mathrm{Wm}}^{-1}K^{-1}$ in the longitudinal direction and of the order of 2.5 to 5.5 ${\mathrm{Wm}}^{-1}K^{-1}$ in the transverse direction [9,38].

For our modelling purposes, the shell thermal conductivity was taken as a free parameter, which can be different for the in-plane and through-plane conduction in the samples of Reference [19]. The difference in the conductivity may come from the compression moulding processing method applied to produce the samples of Reference [19]. The core-shell microbeads are deformed during compression and this influences interaction of the platelets of the shell differently in different directions, thus making the thermal conductivity of the core-shell beads anisotropic.

With regard to the parameters of the Lewis-Nielsen model, the maximal concentration $\varphi_{\max}$ and the parameter A were assumed to be identical for both directions of conduction. This, in case of the parameter A, is an assumption corresponding to a situation where the percolation network develops similarly in both directions. The thermal conductivity of the epoxy matrix was set at 0.25 ${\mathrm{Wm}}^{-1}K^{-1}$, as indicated in the conductivity plots of Reference [19].

**Figure 11:**
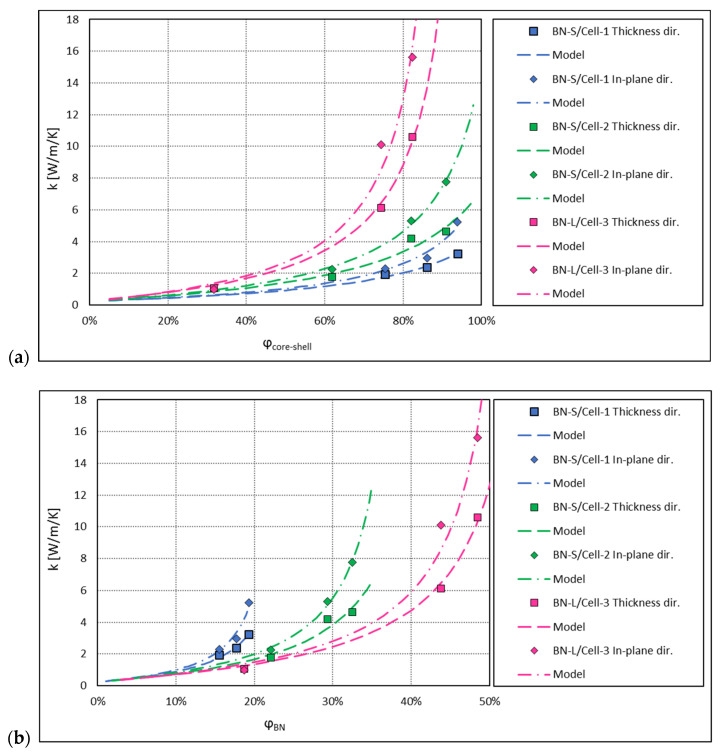
Thermal conductivity of composites with cellulose-BN core-shell fillers of Reference [19]. Lines show the results of model calculations, symbols are measured values: (**a**) Thermal conductivity vs. the core-shell filler volume content in the system, as calculated according to Equation (6). (**b**) Thermal conductivity vs. the absolute volume fraction of BN in the system, as originally presented in Reference [19].

**Figure 12:**
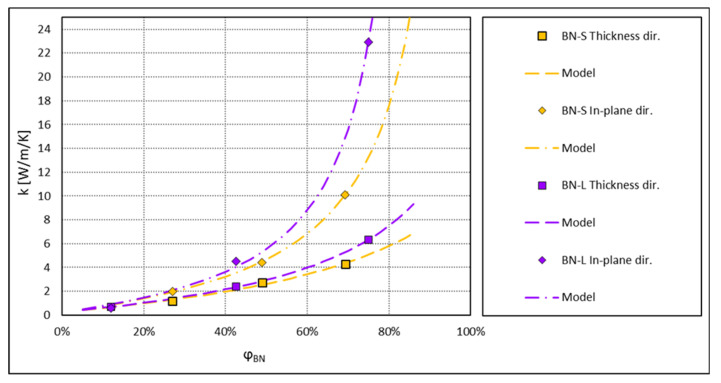
Thermal conductivity of composites with pristine h-BN platelets of Reference [19]. Lines show the results of model calculations, symbols are measured values.

The results of the model calculation compared to the measured thermal conductivities are shown in Figure 11. For completeness, in Figure 12, the model calculation results for the composites with pristine h-BN platelets are shown compared to the measured values obtained in Reference [19]. In all the cases, the model curves fit the experimental points remarkably well. The fit parameters are listed in Table 3 and Table 4 for the core-shell and for the pristine h-BN filler, respectively. When fitting the data, the values of $k_{\mathrm{shell}}$ were limited at $400\;W\;m^{-1}K^{-1}$, and where this value was obtained, it can be interpreted that the effective shell conductivity is close to that of the pure h-BN in the longitudinal direction.

The obtained results are quite consistent. The derived BN-S shell conductivity is of 20 to 25 ${\mathrm{Wm}}^{-1}K^{-1}$ for the measurements in the thickness direction and of 30 to 50 ${\mathrm{Wm}}^{-1}K^{-1}$ for the in-plane direction. This latter value coincides well with the fitted in-plane filler conductivity in the pristine BN-S composite. The values of A of between 3 and 6 for the BN-S core-shell fillers, significantly larger than the Lewis-Nielsen theoretical value for random, not overlapping spheres, indicate that the filler grains are not fully randomly distributed in this kind of composite. They most probably tend to agglomerate into objects of higher aspect ratio. As mentioned before, a similar interpretation of large values of parameter A was given in Reference [7]. In the case discussed here, this may happen due to the compression moulding process, where the core-shell beads interact strongly in a high-shear-rate flow of the moulded material.

For the pristine BN-S composite, even a larger value of A, about 20, was obtained. This is partly due to the platelet nature of the filler with high aspect ratio. For randomly distributed platelets, the theoretical value of that parameter is $A=\sqrt{3}\ln\left(L/l\right)\;$ [26]. As was shown in Reference [27], the resulting parameter A for a typical, randomly oriented h-BN platelet filler, can reach a value of ca. 7.5. The 2 to 3 times higher value obtained here indicates a distribution of angular orientation of the platelets significantly deviating from a random one. This may also be caused by the high-shear-rate flow and interactions between the platelets in the compression moulding process.

Interestingly, for both the core-shell fillers and for the pristine h-BN fillers, the experimental data is very well fitted with the parameter A of identical value for the in-plane and the thickness conduction directions. It was checked that freeing this parameter does not improve the fit, with the result for A being similar in both directions anyway.

For the composite samples with pristine h-BN, this may seem to contradict the common understanding of the Lewis-Nielsen model. In an idealized, theoretical-oriented filler case, e.g., oriented rods, the parameter A is very high in the longitudinal direction and very low in the transverse direction [26]. This is, however, only the case when the filler particles are exactly parallel to each other, which normally does not happen in a realistic system. Moreover, this refers to fillers of isotropic conductivity. For the h-BN platelets, in which the longitudinal thermal conductivity is some 100 times higher than the transverse one, a good thermal contact of one side of the platelet made only at its edge can efficiently deliver heat to its whole surface. Subsequently, the heat may be transferred through the thickness of the platelet to its other side, where it may contact the next one. In such a way, when with the rising concentration of the filler a percolation network is being created by the platelets contacting each other only locally, the effective thermal conductivity of the composite starts to rise equally fast in the longitudinal and in the transverse directions with respect to the oriented filler. This is reflected by the same value of A recorded in both directions. The difference in the conductivity comes from the intrinsic difference of the thermal conductivity in both directions within the platelet, which is reflected in our fitted conductivity values for the pristine BN composites.

In a similar way, for the core-shell fillers, with the very large conductivity of the shell, the thermal contact at an arbitrary point of the shell distributes the heat over the whole surface. In that way, it is not probable to produce an asymmetric percolation network, which would connect the filler grains more efficiently in one direction than the other, hence the unidirectional parameter A and the difference in composite conductivity coming only from the anisotropy of the thermal conductivity of the shell, generated by some squeezing of the microbeads in processing.

The maximum concentration $\varphi_{\max}$ is close to 100%, which is also a result of the compression moulding process, where the core-shell microbeads can deform, thus allowing for extremely dense packing. For the pristine BN-S, the maximum concentration is of around 87%, which fits between the theoretical values for uniaxial random and uniaxial hexagonal close packing of rods or fibres quoted in Reference [26], values well applicable also for platelets.

Similar consistency can be found in the results for the BN-L-based fillers. The in-plane shell conductivity of 400 ${\mathrm{Wm}}^{-1}K^{-1}$ in the core-shell filler coincides with that of the pristine filler. The Lewis-Nielsen parameters for the pristine BN-S and BN-L fillers are very consistent with each other, indicating similar structures being formed in both composites.

The effective filler conductivities of the pristine h-BN fillers in the thickness direction of between 8 and 11 are quite small compared to any other values of the fitted filler conductivities. This indicates to the platelets being quite strongly aligned and, thus, the conduction in the thickness direction being governed largely by the low transverse conductivity of h-BN, of a few ${\mathrm{Wm}}^{-1}K^{-1}$, consistent with the results obtained.

## 4. Conclusions

A model to calculate thermal conductivity of composites with core-shell type fillers was developed based on the Lewis-Nielsen formula for the filled systems and the Russel model of thermal conductivity of porous materials. It was shown that the Russel model, originally developed for a continuous porous material, can also be used to describe the effective thermal conductivity of a single core-shell filler grain. The results of the model calculations were compared with our experimental measurements made on epoxy-based composites with silver-coated glass beads and with bare glass beads used as a reference. In both cases, silane-treated and untreated beads were used. The model was also compared with measurements published by Nagaoka et al. in Reference [19], made on composites filled with cellulose-core microbeads with a h-BN shell and reference composites filled with pristine h-BN.

The main conclusions from the measurements and the model comparison are as follows:

- The Lewis-Nielsen model is an efficient tool to analyse the relation between the filler nature and its arrangement in the composite and the thermal conductivity; with the model developed in this paper, the applicability of this analysis is extended into the area of the core-shell fillers.
- The right approach when applying the Lewis-Nielsen model is to fit both its parameters, with the values limited to the allowed theoretical range, and not to apply the theoretical values as such, based only on the shape of the filler grains. This approach allows to better understand the thermal conductivity mechanisms in the particular example of the composite, such as the effects of aggregation of filler grains into larger structures or the influence of the processing characteristics.
- For spherical fillers, deviations of the parameter A of the Lewis-Nielsen model from the theoretical values can be explained either by a broad distribution of sizes of the filler particles (leading to smaller A) or by aggregation of the particles, forming objects of higher aspect ratio (leading to larger A).
- An oriented mutual arrangement of densely packed h-BN platelets together with the highly anisotropic nature of thermal conductivity of h-BN can lead to an equal value of the parameter A governing conductivity in the transverse and the longitudinal directions. This actually means an enhancement of the thermal conductivity in the direction perpendicular to the platelets compared to what would be expected from merely using the theoretical values of A.
- The parameter A is governed both by the deviation from sphericity of the filler (shape and aspect ratio) and by deviations from randomness of positions and angular orientation of the filler particles. This is the main weak point of the analysis applied, as these two effects cannot be fully distinguished from each other.

Future research should be directed towards defining experimental cases in which the effect of the varying randomness of positions and orientation of the filler grains could be controlled separately from the effect of varying of the aspect ratio of their shape. This could allow to check for the situations where these two effects affect the filler concentration dependence of the thermal conductivity in a measurably different way. One possibility to make this happen would be to apply directed flow of a resin filled with a platelet filler in a mould during hardening of the samples. By varying the flow speed, a different level of orientation of the platelets could be obtained. A subsequent challenging task would be to define a suitable modification of the Lewis-Nielsen model to make it capable to account for the two effects in a separable way.

## Figures and Tables

**Table 1:** Thermal conductivity results obtained at room temperature.

Filler Type	Surface Treatment	Filler Content (vol.%)	$\mathrm{Thermal}\;\mathrm{Conductivity}\;(Wm^{-1}K^{-1})$	Increase with Respect to Reference Filler
reference	no	31%	0.51	-
core-shell	no	31%	0.67	31%
reference	yes	31%	0.48	-
		45%	0.52	-
core-shell	yes	31%	0.85	77%
		45%	0.96	84%

**Table 2:** Lewis-Nielsen model parameters of the plots shown in Figure 8 and Figure 9.

Filler	*A*	*φ*_max_ (vol.%)
Untreated filler	1.50	42.0%
Silane-treated filler	4.07	74.1%

**Table 3:** Model parameters of the plots shown in Figure 10. The fitted parameters are A, $\varphi_{\max}$, and $k_{\mathrm{shell}}$, while $k_{\mathrm{eff}\;\mathrm{grain}}$ is calculated according to Equation (4). BN-S and BN-L refer to the filler shell material made of 8 and 18 μm boron nitride platelets respectively. Cell-1, Cell-2, and Cell-3 refer to cellulose beads making the core of the filler of diameters 30, 40, and 35 μm respectively. For details refer to Reference [19].

Core-Shell Filler	A	$\varphi_{\max}(\mathrm{vol}.\%)$	Thickness Direction		In-Plane Direction	
			$k_{\mathrm{shell}}\;$ $\left(Wm^{-1}K^{-1}\right)$	$k_{\mathrm{eff}\;\mathrm{grain}}$ $\;\left(Wm^{-1}K^{-1}\right)$	$k_{\mathrm{shell}}$ $\;\left(Wm^{-1}K^{-1}\right)$	$k_{\mathrm{eff}\;\mathrm{grain}}$ $\;\left(Wm^{-1}K^{-1}\right)$
BN-S/Cell-1	3.46	93.9%	21.1	3.41	32.7	5.17
BN-S/Cell-2	6.01	100%	24.8	7.23	51.9	14.9
BN-L/Cell-3	8.51	92.1%	54.8	28.6	400	208

**Table 4:** Lewis-Nielsen model parameters of the plots shown in Figure 11. BN-S and BN-L refer to the hexagonal boron nitride (h-BN) platelets of the size of 8 and 18 μm, respectively, see Reference [19].

Pristine h-BN Filler	A	$\varphi_{\max}\;(\mathrm{vol}.\%)$	Thickness Direction	In-Plane Direction
			$k_{f}$ $\left(Wm^{-1}K^{-1}\right)$	$k_{f}$ $\left(Wm^{-1}K^{-1}\right)$
BN-S	21.1	86.9%	8.03	33.1
BN-L	18.8	86.9%	11.0	400
